# Assessment of angiotensin-converting enzyme inhibitor/angiotensin receptor blocker on the split renal function in the patients with primary hypertension

**DOI:** 10.1097/MD.0000000000025928

**Published:** 2021-05-21

**Authors:** Jingsi Zhang, Mingyu Wang, Kehui Sun, Yanchun Ding

**Affiliations:** Department of Cardiology II, the Second Hospital of Dalian Medical University, Dalian, China.

**Keywords:** ACEI/ARB, primary hypertension, renal dynamic imaging, split renal function

## Abstract

Bilateral kidney damage in hypertensive patients is not parallel. Angiotensin-converting enzyme inhibitor/angiotensin receptor blocker (ACEI/ARB), as a commonly used antihypertensive drug, could protect kidney function and delay its deterioration. Most studies focused on overall renal function, but the researches on split renal function (SRF) are rare. We investigated the effects of ACEI/ARB on the SRF in patients with primary hypertension.

Patients with primary hypertension (n = 429; male: 213; female: 216) admitted to our department between January 2014 and December 2016 were included in this study. The glomerular filtration rate (GFR) of split and total renal function were determined using diethylenetriaminepentaacetic acid tagged with 99mTc renal dynamic imaging method. For the same patient, the side with high GFR was considered as higher GFR kidney, whereas that with a low GFR was considered as lower GFR kidney. The split function score (*Q* value) was utilized to evaluate the differences of bilateral renal function. The patients were divided into 3 groups based on the *Q* values (Group 1, *Q* value <5%; Group 2, *Q* value of 5%–10%; Group 3, *Q* value ≥10%). All the patients received antihypertensive therapy based on ACEI/ARB. The renal dynamic imaging was performed in the 1-year follow-up to investigate the changes of the SRF.

Compared with the baseline level, significant decline was noticed in the serum creatinine (Scr) in Group 2 and Group 3 (*P* < .05). The cystatin C in Group 3 showed significant decline (*P* < .05). Compared with the baseline, there was significant decline in the Q value in Group 2, whereas the GFR of lower GFR kidney showed significant increase (*P* < .05). No statistical differences were noticed in the *Q* value and split GFR in Group 1 and Group 3 (*P* > .05).

In primary hypertension patients, ACEI/ARB therapy could improve the SRF of lower GFR kidney in the presence of certain differences between the SRF. As a result, the SRF difference was reduced. In case of *Q* value in a range of 5% to 10%, ACEI/ARB could improve the renal function effectively. It may be significant for the design of antihypertensive drugs.

## 1 Introduction

Renal injury is a common complication of primary hypertension.^[1]^ Renin-angiotensin-aldosterone system (RAAS), including local and circulating RAAS, plays important roles in the blood pressure regulation and the pathogenesis of chronic renal injury.^[2]^ Juxtaglomerular cells could secrete rennin, and then activate the angiotensinogen and lead to generation of angiotensin-I (AT-I).^[3]^ In pulmonary vascular endothelial cells, AT-I was transmitted to angiotensin-II (AT-II) by angiotoninase (ACE). The AT-II would result in increase of blood volume through directly involving in the constraction of the arteriola or stimulating the secretion of aldosterone in the glomerular zone of adrenal cortex.^[4,5]^ Alternatively, it could obviously increase the blood pressure through promoting the release of catecholamine by the adrenal medulla and the sympathetic nerve ending.^[6]^ To our best knowledge, AT-II was associated with the development of glomerular hypertension,^[7]^ which then may contribute to the hemodynamic changes. This process is reported to be related to the elevation of glomerular capillary pressure, proteinuria progression, and glomerulosclerosis.^[8]^ In addition, the progression of the proteinuria would further lead to deterioration of the renal injury. The protein exposure of tubular cells would trigger the release of the proinflammatory cytokines, together with the cellular aggregation and even subsequent injury.^[9]^ On this basis, angiotensin-converting enzyme inhibitors (ACEI) and angiotensin II receptor blockers (ARB) have been commonly utilized in clinical settings to modulate blood pressure and protect renal function.

As the major RAS inhibitor, ACEI/ARB could effectively bring down the systemic hypertension, glomerular hypertension, as well as proteinuria level.^[10]^ In addition, it could inhibit the cellular proliferation and hypertrophy, and deduce the release of cytokines and other chemokines. Moreover, it could attenuate the accumulation of extracellular matrix in the glomerular cells and delay the progression of the renal fibrosis.^[11]^ Nevertheless, the efficiency of ACEI/ARB in dilating the efferent arteriole of glomerulus was superior to that of the afferent arteriole of glomerulus, which then resulted in the decline of glomerular filtration pressure and renal function, as well as glomerular filtration rate (GFR).^[12]^ In the early stage, ACEI/ARB may trigger the elevation of serum creatinine (Scr) and potassium concentration.^[13]^ According to the previous description, part of the hypertensive patients may present unpaired renal damages.^[14]^ Nevertheless, rare studies have focused on the split renal function (SRF) of the patients after long-term administration of ACEI/ARB, as well as the alteration in the difference between the lower GFR and higher GFR kidney. The clearance rate of isotope-labeled substances is considered the criterion standard for the evaluation of GFR, with diethylenetriaminepentaacetic acid tagged with 2WQ299mTc (99mTc-DTPA) commonly utilized in clinical settings. Such method is effective with good reproducibility. In this study, 99mTc-DTPA renal dynamic imaging method was utilized to determine the total and split GFR, with an aim to investigate the effects of ACEI/ARB on the SRF in the hypertensive patients.

## 2 Materials and methods

### 2.1 Subjects

Patients with primary hypertension admitted to our department between January 2014 and December 2016 were included in this retrospective study. The diagnosis of hypertension was performed based on the criteria proposed by the Chinese Medical Association.^[15]^ The hypertension diagnosis was based on the following standards: presence of systolic blood pressure (SBP) ≥140 mmHg and/or diastolic blood pressure (DBP) ≥90 mmHg on different 3 days without administration of antihypertensive drugs. The exclusion criteria were as follows: those with secondary hypertension, diabetes mellitus, primary renal disease, gout, urinary system infection, or malignancy; those with allergy or without tolerance to the ACEI/ARB; those with severe heart failure, New York Heart Association class III-IV; those with severe cerebral or hepatic disease; or those with renal artery stenosis after ultrasonic examination. Patients presenting severe renal insufficiency with Scr of higher than 265 μmol/L were not included in this study. This study waived the Ethical approval as it was a retrospective analysis. Only the clinical data of patients were collected without intervention in the treatment protocols. There was no physiological risk to patients, and the privacy of patients was not disclosed. Therefore, informed consent was not given in this study.

### 2.2 Grouping

The SRF difference (*Q* value) was used to evaluate the differences between both sides of kidney of the same patient. The patients were divided into 3 groups according to the *Q* value, Q value of <5% (Group 1), 5% ≤*Q* value <10% (Group 2) and ≥10% (Group 3), respectively. For the same patient, the side with high GFR was considered as higher GFR kidney, whereas that with a low GFR was considered as lower GFR kidney. All the patients received renal dynamic imaging again in the 1-year follow-up.

### 2.3 Methods

All the subjects included in this study received antihypertensive therapy based on ACEI/ARB, and then the patient characteristics including age, sex, case history, blood pressure, and body mass index were collected. Within 24 hours after admission, we determined the fasting blood glucose (FBG), total cholesterol, triglyceride, high-density lipoprotein cholesterol, low-density lipoprotein cholesterol, alanine aminotransferase, aspartate transaminase, albumin, Scr, urea nitrogen (UREA), Cystatin C (CysC), and uric acid (UA) after 8 hours of fasting. The indices were measured using the automatic biochemistry analyzer (Hitachi 7600A, Tokyo, Japan) and the ADVIA 2400 biochemical analyzer (Simens, Berlin, Germany). The commercial kits utilized for the determination of FBG, blood fat, liver function, Scr, and UA were purchased from the LeadMan (Peking, China). The CysC was measured using the immune colloidal gold technique.

The renal dynamic 99mTc-DTPA was monitored using the Millennium Hawkeye VG Imaging facility (GE Healthcare, CA). The 99mTc-DTPA was administrated into the right median cubital vein in a bolus pattern. Single photon emission computed tomography was utilized for the dynamic imaging collection, followed by depicting the areas of renal and abdominal aorta. On this basis, the GFR of the total and split kidney were calculated together with the parameters related to the blood perfusion of kidney and the renal functional curvature. In addition, the renal morphology and function and the drainage of urinary tract were monitored.^[16]^ The GFR of the split kidney was monitored using the 99mTC-DTPA, to calculate the QR, QL, and Q, respectively. *Q* value was calculated based on the following formula as previously described^[17]^: Q_L_ = GFR_L_/GFR_R_ + L; Q_R_ = GFR_R_/GFR_R_ + L; Q = |QL–QR|.

### 2.4 Statistical analysis

SPSS 22.0 software was used for the statistical analysis (SPSS Inc, Chicago, IL). Measurement data were tested for the normal distribution. The data that were normally distributed were presented as mean ± standard deviation. Student *t* test was performed for the inter-group comparison. Analysis of variance was conducted for the multigroup comparison. For the data with significant differences after multigroup comparison, LSD method was used for the comparison. The data that were not normally distributed were tested using the nonparametric test and were presented as median. *P* < .05 was considered to be statistically significant.

## 3 Results

### 3.1 Patient characteristics

There were no statistical differences in the age, sex, body mass index, SBP, DBP, alanine aminotransferase, aspartate transaminase, albumin, total cholesterol, triglyceride, high-density lipoprotein cholesterol, low-density lipoprotein cholesterol, and FBG (*P* > .05). Besides, there were no statistical differences in the treatment regimen of ACEI/ARB among the 3 groups (*P* > .05, Table 1).

**Table 1:** Patient characteristics of the 3 groups.

	Group 1 (n = 209)	Group 2 (n = 132)	Group 3 (n = 77)	*χ*^2^	*P*
Age, y	66.64 ± 16.10	70.14 ± 13.58	67.10 ± 13.54	0.148	.863
Male/female	107/113	61/71	45/32	0.806	.369
BMI, kg/m^2^	26.99 ± 2.47	26.70 ± 3.29	27.50 ± 4.30	0.123	.885
SBP, mmHg	155.40 ± 15.49	154.73 ± 13.91	154.45 ± 15.11	0.538	.591
DBP, mmHg	90.20 ± 8.44	90.88 ± 8.71	85.09 ± 9.54	2.226	.129
ALT, U/L	25.18 ± 9.21	31.29 ± 14.53	25.17 ± 15.56	0.570	.573
AST, U/L	21.82 ± 3.60	31.43 ± 17.37	21.80 ± 7.61	2.467	.105
ALB, g/L	43.83 ± 3.90	43.40 ± 4.33	43.636 ± 6.87	0.14	.986
TC, mmol/L	5.34 ± 1.28	4.89 ± 1.26	4.97 ± 1.20	0.362	.700
TG, mmol/L	4.89 ± 1.26	3.44 ± 4.18	1.56 ± 0.614	1.102	.348
HDL-C, mmol/L	1.02 ± 0.17	1.19 ± 0.29	1.23 ± 0.32	1.877	.174
LDL-C, mmol/L	2.98 ± 0.85	1.20 ± 0.80	2.91 ± 1.01	2.918	.073
FBG, mmol/L	6.99 ± 2.81	6.04 ± 1.73	6.24 ± 2.07	0.446	.645
ACEI/ARB	75/134	43/89	26/51	0.734	.392

### 3.2 Comparison of Scr, UREA, CysC, UA, and blood pressure

Before treatment, the Scr and CysC in Group 3 were significantly higher than those in Group 1 and Group 2 (*P* < .05). In the 1-year follow-up, there were no obvious changes in the UREA compared with the baseline line in 3 groups (*P* > .05). After treatment, the Scr and CysC showed significant decline compared with the baseline levels, especially in Group 3 (*P* < .05). Compared with the baseline level, significant decline was noticed in the SBP and DBP in Group 1, Group 2 and Group 3, especially Group 3 (*P* < .05, Table 2).

**Table 2:** Comparison of UREA, Scr, CysC, and UA of the 3 groups.

	Group 1 (n = 209)		Group 2 (n = 132)		Group 3 (n = 77)	
	Baseline	Post-treatment	Baseline	Post-treatment	Baseline	Post-treatment
UREA, mmol/L	6.77 ± 1.56	6.05 ± 1.69	5.87 ± 1.11	5.82 ± 1.79	8.39 ± 4.74	7.46 ± 2.82
Scr, μmol/L	72.8 ± 24.06	71.2 ± 18.23	69.90 ± 16.47	60.26 ± 23.33	111.29 ± 60.34^∗^^Δ^	90.20 ± 43.43^▿^
CysC, mg/L	1.06 ± 0.24	1.05 ± 0.25	1.10 ± 0.34	1.10 ± 0.23	1.59 ± 0.80^∗^^Δ^	1.39 ± 0.65^▿^
UA, μmol/L	365.53 ± 63.50	378.34 ± 136.12	345.46 ± 93.75	329.58 ± 67.40	379.37 ± 106.67	382.48 ± 148.58
SBP, mmHg	155.40 ± 15.49	129.70 ± 7.54^▿^	154.73 ± 13.91	133.13 ± 5.35^▿^	154.45 ± 15.11	136.45 ± 8.43^▿^
DBP, mmHg	90.20 ± 8.44	83.30 ± 1.07^▿^	90.88 ± 8.17	79.50 ± 5.35^▿^	85.09 ± 9.54	81.36 ± 5.33

### 3.3 Comparison of SRF

Among the patients with primary hypertension, the bilateral renal injury was different. Before treatment, there were statistical differences in split renal GFR of the same patient (*P* < .05). After treatment, no statistical differences were noticed in the split renal GFR compared to baseline (*P* > .05, Table 3).

**Table 3:** Comparison of GFR in the lower GFR kidney and higher GFR kidney.

GFR	Baseline	Post-treatment
GFR (mL/min), higher GFR side	41.60 ± 16.00	41.89 ± 13.68
GFR (mL/min), lower GFR side	34.77 ± 14.90^∗^	35.18 ± 13.72

### 3.4 Comparison of split renal GFR and *Q* value in each group

Compared with the baseline level, significant decline was noticed in the SRF difference in Group 2 after 1-year treatment, represented by the decline of Q value. Significant elevation was noticed in the GFR of the lower GFR kidney (*P* < .05). In Group 1 and 3, there were no statistical differences in the *Q* value and split renal GFR compared with the baseline levels (*P* > .05, Table 4).

**Table 4:** Comparison of Q value and renal function of each group.

	Group 1 (n = 209)		Group 2 (n = 132)		Group 3 (n = 77)	
	Baseline	Post-treatment	Baseline	Post-treatment	Baseline	Post-treatment
*Q* value (%)	2.31 ± 1.63	1.93 ± 4.90	8.30 ± 1.21	4.90 ± 4.16^∗^	18.12 ± 9.43	16.98 ± 11.51
GFR (mL/min), higher GFR side	43.50 ± 16.33	44.08 ± 13.21	37.41 ± 18.19	40.16 ± 12.45	41.38 ± 17.52	41.71 ± 13.07
GFR (mL/min), lower GFR side	41.80 ± 15.62	42.09 ± 12.79	31.80 ± 15.71	37.05 ± 13.21^∗^	30.27 ± 14.77	30.52 ± 12.06

## 4 Discussion

Early-stage hypertension may induce multiple-organ damages, among which renal dysfunction is featured by late onset and rapid progression. Finally, it may lead to renal failure and even death, which receives more attention. According to a previous study, the incidence of renal dysfunction caused by primary hypertension was only inferior to the cardiac complications, among which a large number of cases died from renal failure.^[18]^ Renal pathological changes resulted from hypertension were mainly featured by lesions in the renal arterioles, which then subsequently resulted in ischemic changes in the glomerulus, as well as renal dysfunction and failure.^[19]^ In hypertensive patients, the decline of the blood perfusion in 2 kidneys might not be symmetric, which might trigger the unpaired renal function damages.^[20]^ To our best knowledge, rare studies have focused on the evaluation of split renal dysfunction induced by hypertension.^[21]^ In a previous study, Schutten et al included 146 cases with primary hypertension, and then the rennin and aldosterone levels of both sides were determined together with the renal blood flow. The study demonstrated that the mean blood flow in the left kidney in the primary hypertension patients was significantly lower than that of the right kidney. In addition, there was a negative correlation between renal blood flow and aldosterone-renin ratio. This indicated that there might be differences in the renal damages between both kidneys among the hypertensive patients.^[22]^ An animal experiment indicated that the distribution of the sympathetic nerves in both kidneys was different. Hypoxia could lead to sympathetic activation, which further resulted in renal fibrosis and reduced renal blood flow. Therefore, the level of fibrosis in bilateral kidneys was not parallel.^[23]^ For the patients with unilateral renal artery stenosis, functional renal impairment was usually not clinically detectable because of the compensatory function of the intact contralateral kidney. The renal dynamic imaging contributed to the evaluation of SRF. Therefore, it has been widely used in the diagnosis of unilateral renal artery stenosis.^[24]^ In this study, such method was used to determine the SRF, which showed that in patients with primary hypertension, the GFR of one kidney was significantly higher than that of the other, and the damage of bilateral kidneys was not parallel. This was in line with the previous studies.

ACEI, a common antihypertensive drug, has been widely used in treating diabetes mellitus, heart failure, and coronary heart disease.^[25]^ In a previous study, the GFR of the patients with primary hypertension complicated with renal dysfunction received long-term ACEI administration showed significant increase, especially in those with lower renal perfusion pressure, but autoregulating function was still available.^[26]^ Afterwards, AASK study investigated the outcome of hypertensive renal dysfunction; ramipril was superior to the combination of amlodipine and metoprolol in the views of complicated outcome in the end-stage renal disease.^[27]^ AIPRI study validated that ACEI could reduce the risk of renal failure.^[28]^ In pharmaceutical views, ACEI could act on RAAS, inhibit the transmission of AT-I into AT-II, which induced the decline of AT-II concentration. Besides, it could inhibit the degradation of the bradykinin, which then played protective roles on the renal function. As a new antihypertensive agent, ARB is commonly accepted by the patients compared to ACEI as it induces less dry cough, dry mouth, and headache. In IDNT study performed in 2000, the risk of Scr multiplication in the Irbesartan group was significantly lower than that of the placebo group.^[29]^ In pharmaceutical views, ARB could bind with the angiotensin type 1 receptor in a competitive manner, which then deduced the activity of AT-II. Meanwhile, it could contribute to the synthesis of bradykinin and play protective roles. In summary, ACEI/ARB could block the RAAS, and then led to dilatation of renal artery and increase of renal blood flow.^[28]^ In addition, ACEI/ARB could decrease the glomerular filtration pressure, reduce the protein filtration through the glomerular capillaries, inhibit the excessive accumulation of extracellular matrix, and slow down the development of glomerular sclerosis. Many clinical studies confirmed that ACEI/ARB showed important therapeutic potential for patients with chronic renal insufficiency. A large cohort study showed that ACEI/ARB significantly reduced all-cause mortality in nondialysis patients with chronic renal insufficiency.^[30]^ Maione et al showed that ACEI significantly reduced the risk of non-fatal cardiovascular events. ACEI/ARB could also significantly decrease the incidence of end-stage renal disease and proteinuria.^[31]^ In the proposed guidelines of hypertension, ACEI/ARB has been recommended in clinical practices for hypertension combined with chronic renal diseases. ACEI/ARB could control blood pressure and protect kidney by blocking RAAS, and the renal damage in hypertension is not parallel. Thus, we speculated that ACEI/ARB may affect the SRF. However, rare studies have focused on the efficiency of long-term application of ACEI/ARB on the function of split kidney.

In this study, we compared the concentrations of CysC and Scr in the 3 groups and found that the Scr and CysC levels declined, especially in Group 3 after treatment. The result indicated that ACEI/ARB therapy could delay renal function deterioration or even improve renal function, especially in those with *Q* value ≥10%. However, no statistical differences were noticed in split renal GFR in all cases after treatment compared with the baseline levels. The possible reason is that the therapeutic effect of ACEI/ARB was different in the 3 groups. We further grouped based on *Q* value, and analyzed the improvement effect of ACEI/ARB on GFR when *Q* values were in different ranges. Results showed that in cases of Q value in a range of 5% to 10%, ACEI/ARB could decline the functional differences of the split kidney, together with improving the GFR in the lower GFR kidney. In the presence of a *Q* value of ≥10 or <5, these effects were not obvious. These results indicated that when the SRF difference increased gradually, ACEI/ARB could protect the renal function effectively. However, the protection effect of ACEI/ARB declined when the renal function difference increased to a certain extent.

There are some limitations in this study. We only focused on the hypertension patients those with creatinine level of <265 μmol/L. In future, further studies are required to investigate the efficiency of ACEI/ARB treatment on the SRF in the patients with severe renal insufficiency.

In summary, renal dynamic imaging is a commonly used technique to evaluate GFR and detect renal artery stenosis. Although it could also be used to evaluate the SRF difference (*Q* value), *Q* value has not been widely used in clinical practice, and studies on *Q* value are very few. Our study showed that ACEI/ARB could effectively improve GFR of lower GFR kidney in patients with primary hypertension when the *Q* value was within a certain range. Therefore, in cases of *Q* value in a range of 5% to 10%, ACEI/ARB would be a better choice to improve the renal function. It is of great value to the selection of antihypertensive drugs.

## Author contributions

ZJS wrote the manuscript; DYC revised the manuscript; WMY, SKH did the data analysis and the data collection.

**Conceptualization:** Mingyu Wang.

**Data curation:** Mingyu Wang.

**Formal analysis:** Mingyu Wang.

**Investigation:** Kehui Sun.

**Methodology:** Kehui Sun.

**Resources:** Kehui Sun.

**Writing – original draft:** Jingsi Zhang.

**Writing – review & editing:** Yanchun Ding.
